# Dynamic Stabilization Adjacent to Fusion versus Posterior Lumbar Interbody Fusion for the Treatment of Lumbar Degenerative Disease: A Meta-Analysis

**DOI:** 10.1155/2020/9309134

**Published:** 2020-05-20

**Authors:** Xiangyao Sun, Zhaoxiong Chen, Siyuan Sun, Wei Wang, Tongtong Zhang, Chao Kong, Shibao Lu

**Affiliations:** ^1^Department of Orthopaedics, Xuanwu Hospital Capital Medical University, Beijing 100053, China; ^2^National Clinical Research Center for Geriatric Diseases, Beijing 100053, China; ^3^Charité-Universitätsmedizin Berlin, Berlin 113353, Germany; ^4^Department of Interdisciplinary, Life Science, Purdue University, West Lafayette, IN 47907, USA; ^5^Department of Orthopaedics, Chui Yang Liu Hospital affiliated to Tsinghua University, 100020 Beijing, China

## Abstract

This study evaluated differences in outcome variables between dynamic stabilization adjacent to fusion (DATF) and posterior lumbar interbody fusion (PLIF) for the treatment of lumbar degenerative disease. A systematic review of PubMed, EMBASE, and Cochrane was performed. The variables of interest included clinical adjacent segment pathologies (CASPs), radiological adjacent segment pathologies (RASPs), lumbar lordosis (LL), visual analogue scale (VAS) of back (VAS-B) and leg (VAS-L), Oswestry disability index (ODI), Japanese Orthopaedic Association (JOA) score, duration of surgery (DS), estimated blood loss (EBL), complications, and reoperation rate. Nine articles identified as meeting all of the inclusion criteria. DATF was better than PLIF in proximal RASP, CASP, and ODI during 3 months follow-up, VAS-L. However, no significant difference between DATF and PLIF was found in distal RASP, LL, JOA score, VAS-B, ODI after 3 months follow-up, complication rates, and reoperation rate. These further confirmed that DATF could decrease the proximal ASP both symptomatically and radiographically as compared to fusion group; however, the influence of DATF on functional outcome was similar with PLIF. The differences between hybrid surgery and topping-off technique were located in DS and EBL in comparison with PLIF. Our study confirmed that DATF could decrease the proximal ASP both symptomatically and radiographically as compared to the fusion group; however, the influence of DATF on functional outcome was similar with PLIF. The difference between hybrid surgery and topping-off technique was not significant in treatment outcomes.

## 1. Introduction

Low back pain (LBP) is one of the most important reasons for seeking medical treatment with a prevalence ranging from 59% to 84% [1]. Lumbar degenerative diseases, such as lumbar spinal stenosis, lumbar disc herniation, and lumbar degenerative instability, are common etiologies of LBP and can have a significant influence on the quality of life [2]. It is well known that posterior rigid transpedicular stabilization with intervertebral fusion is considered the most widely used treatment for lumbar degenerative disease. However, long-term follow-up confirmed a high incidence of adjacent segment disease (ASD) after fusion surgery, which makes it a problematic treatment method [3]. Dynamic interspinous spacer devices can reduce the stiffness of the instrumentation to preserve more physiological load transmission, which shows a better restoration of the physiological mechanics of the spinal segments [4]. Khoueir et al. [5] defined three types of posterior dynamic stabilization systems: hybrid stabilization device with pedicle screw or rod construct (HSD) such as Dynesys and DTO®; interspinous process devices (IPD) such as X-STOP, Wallis, DIAM, and Coflex; total facet replacement system (TFR).

Hybrid stabilization involves the application of two different kinds of devices, the dynamic stabilization (IPD or PDS) and fusion. It is important to differentiate topping-off and hybrid stabilization, each of which represents very different surgical strategies. The aim of hybrid surgery is to prevent further degeneration of the asymptomatic adjacent segment rather than replace fusion when treating symptomatic degenerated adjacent segment [6]. However, an asymptomatic segment, which would otherwise be left untreated, can be included in topping-off that extends a rigid stabilization system with a dynamic element [7]. Unfortunately, these two concepts were confused in most of the previous studies [3, 8, 9], which could make the results less reliable.

Pan et al. [10] carried out a meta-analysis comparing motion-preservation preservation procedures and fusion in the lumbar spine to evaluate the efficacy of preventing the adjacent segment degeneration (ASDeg) or adjacent segment disease (ASDis). However, they mixed dynamic stabilization alone, hybrid stabilization, and topping-off in their study, which could make the results inaccurate. Chou et al. [11] discussed the effect of topping-off techniques to decrease the occurrence of ASD after lumbar fusion surgery. Similarly, they simply defined the topping-off technique as a concept applying less rigid fixation for the purpose of avoiding ASD, which failed to differentiate topping-off and hybrid stabilization. Therefore, there is an urgent need for a rigorous research to provide more reliable results.

In this study, we conducted a meta-analysis to analyze all available data on postoperative clinical and radiographic parameters of dynamic stabilization adjacent to fusion (DATF) versus posterior lumbar interbody fusion (PLIF) for the treatment of multilevel lumbar degenerative disease. In order to differentiate hybrid stabilization and topping-off technology in the reviewed studies, all the treatment methods and inclusion criteria in these studies were carefully evaluated. The methods mentioned above can make this study more reliable.

## 2. Materials and Methods

### 2.1. Search Strategy

The primary sources for the literature review were PubMed (1950–2019), Embase (1980–2019), and the Cochrane Central Register of Controlled Trials (2019 edition), to identify trials according to Cochrane Collaboration guidelines. The search included articles published up to June 2019 with no lower date limit on search results. This review was conducted in accordance with the Preferred Reporting Items for Systematic Reviews and Meta-Analyses (PRISMA) Statement [12]. The following search terms and different combinations of MeSH (Medical Subject Heading) terms and textual words were used: “hybrid stabilization”, “topping off”, “hybrid stabilization device”, “hybrid fixation”, “dynamic hybrid”, “dynamic”, “fusion”, “lumbar degenerative disease”, “adjacent segment degeneration”, “lumbar”, and “adjacent segment disease”. Manual searches of the reference lists of all included studies were carried out to identify studies that the electronic search may have failed to identify. Two reviewers (XYS and SYS) excluded duplicate results and independently screened studies according to inclusion and exclusion criteria. Any disagreements were resolved by a third researcher (SBL).

### 2.2. Selection Criteria and Quality Assessment

**Figure 1:**
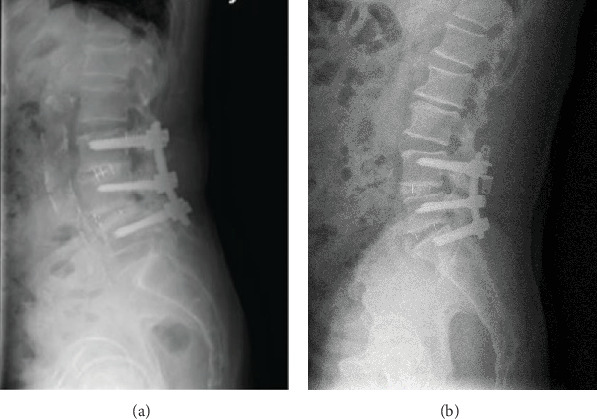
Lateral X-ray of lumbar spine showing (a) PLIF, Pedicle screws were inserted bilaterally at L3–L5 for internal fixation. Interlaminar fenestration or total laminectomy was performed bilaterally at L4/5 for decompression. Interlaminar fenestration was performed bilaterally for decompression at L3/4, with preservation of the lateral 1/2 of the facet joint; (b) hybrid stabilization (Coflex+PLIF), the same procedures were performed to expose the target area and to manipulate the segments at L4–S1 as PLIF. Coflex was inserted to L3/4 with interlaminar fenestration for decompression.

**Figure 2:**
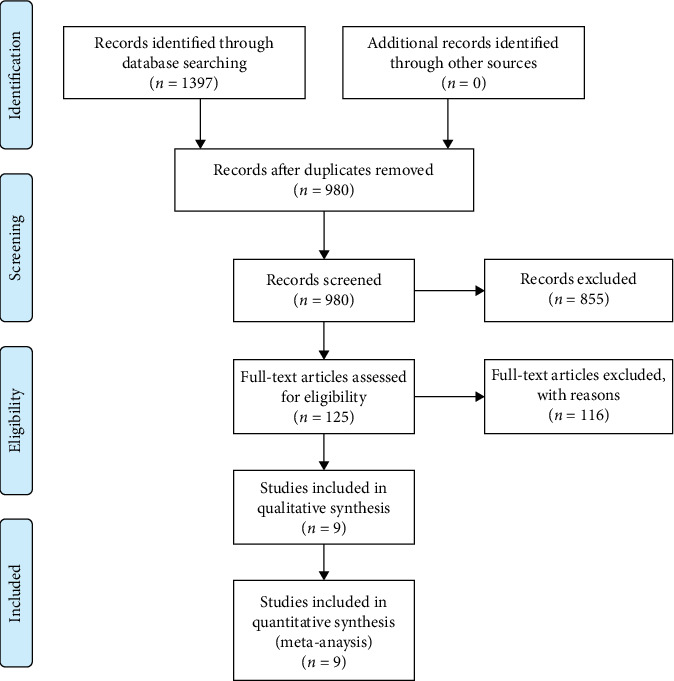
Flow chart showing the identification and selection of cases.

Randomized controlled trials (RCTs), retrospective studies, and prospective studies were selected in this study. Inclusion criteria for this study consisted of the following: (1) patient underwent posterior lumbar interbody fusion (PLIF) or hybrid stabilization for lumbar degenerative disease (Figure 1); (2) in the hybrid surgery, dynamic stabilization system was applied to the symptomatic degenerative segment without spinal instability which was next to the adjacent fusion segment; (3) progression of ADS, complications, and other factors relevant to the postoperative outcomes of the disease were provided in the articles; (4) the follow-up time was more than 12 months; (5) the language of studies was limited to English. We excluded studies where (1) case reports, review articles, comments, letters, biomechanical studies, or animal experiments were performed; (2) the full text of the article was unavailable; (3) duplicate publications were performed; (4) inclusion criteria were not met.

### 2.3. Data Extraction and Quality Assessments

Two investigators (XYS and SYS) independently extracted data from included studies. The following information was carefully extracted from all qualified studies: years of publication, authors, nations and ethnicities of study populations, numbers of cases, clinical adjacent segment pathologies (CASPs) [13, 14], radiological adjacent segment pathologies (RASPs) [13, 15, 16], lumbar lordosis (LL), visual analogue scale (VAS) score of back (VAS-B) and leg (VAS-L), Oswestry disability index (ODI), Japanese Orthopaedic Association (JOA) score, duration of surgery (DS), estimated blood loss (EBL), complications, and reoperation rate. Two reviewers (WW and CK) independently assessed the quality of the included studies according to the Newcastle-Ottawa scale (NOS) [17] and level of evidence (LoE) [18]. The study with a score of 7 or more was considered as an excellent quality study.

### 2.4. Data Analysis

Statistical analysis was performed using STATA version 14.0 (StataCorp LP, College Station, Texas, USA). DATF was set to the experimental group. Then, PLIF was set to control group. Heterogeneity was evaluated using the *I*^2^ statistics and *χ*^2^ test. It was considered significant when *I*^2^ > 50% or *p* value for *χ*^2^ < 0.1. The odds ratios (OR) and 95% confidence interval (CI) were calculated for binary outcomes. Weighted mean differences (WMD) and 95% CI were calculated for continuous outcomes. Random-effect models were applied unless statistical heterogeneity was insignificant, in which case fixed-effect models were used. Through subgroup analysis, the influence of study design and fixed levels on pooled estimates was investigated by us. In addition, the Egger test was used to analyze the publication bias.

## 3. Results

### 3.1. Study Characteristics

The initial PubMed, EMBASE, and Cochrane Review search resulted in 137 articles (Figure 2). This study excludes 116 articles through full-text review. This was because all of these articles did not provide the reviewers with the precise definitions of hybrid stabilization and topping-off technology. Nine [3, 8, 9, 13, 19–23] articles identified as meeting all of the inclusion criteria after two-reviewer assessment. The characteristics of the included studies can be found in Table 1.  Table 2 showed the results of the Newcastle-Ottawa scale (NOS).

Notice: RCT: randomized controlled trial, Retro: retrospective cohort study, Pro: prospective cohort study, RASP: Radiological adjacent segment pathology, CASP: Clinical adjacent segment pathology, VAS: visual analog scale, ODI: Oswestry disability index, JOA: Japanese Orthopaedic Association score, LL: lumbar lordosis, EBL: estimated blood loss, DS: Duration of surgery, NA: not available.

### 3.2. Comparison of the Radiographic Outcomes

**Figure 3:**
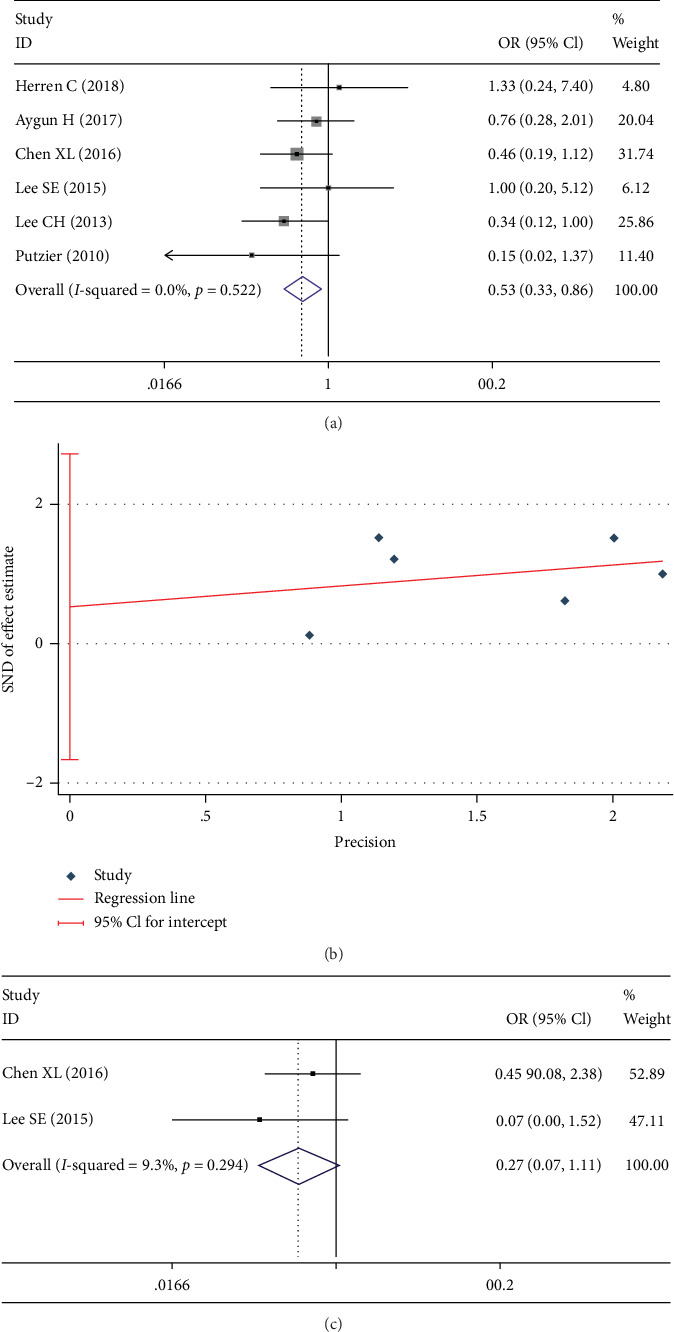
Test results showing (a) forest plot of RASP proximal to lumbar fusion, (b) Egger graph of RASP proximal to lumbar fusion, (c) forest plot of RASP distal to lumbar fusion.

RASP was defined as the imaging changes next to previously operated levels [13, 15, 16]. RASP proximal to lumbar fusion was documented in 7 studies [3, 13, 19–23]. There was a significant heterogeneity between these studies (*I*^2^ = 51.1%). However, this disappeared after the exclusion of one study [20] (*I*^2^ = 0%, Figure 3(a)). The rate of RASP in the DATF group was significantly less than the PLIF group in the fixed-effects model (OR 0.528; 95% CI 0.326, 0.856; *I*^2^ = 0%; *p* = 0.010). Egger test showed that no significant publication bias was found (*p* = 0.532, Figure 3(b)). RASP distal to lumbar fusion was documented in 2 studies [3, 21]. No difference was found in RASP distal to lumbar fusion between DATF group and PLIF group in the fixed-effects model (OR 0.270; 95% CI 0.066, 1.111; *I*^2^ = 9.3%; *p* = 0.070, Figure 3(c)).

**Figure 4:**
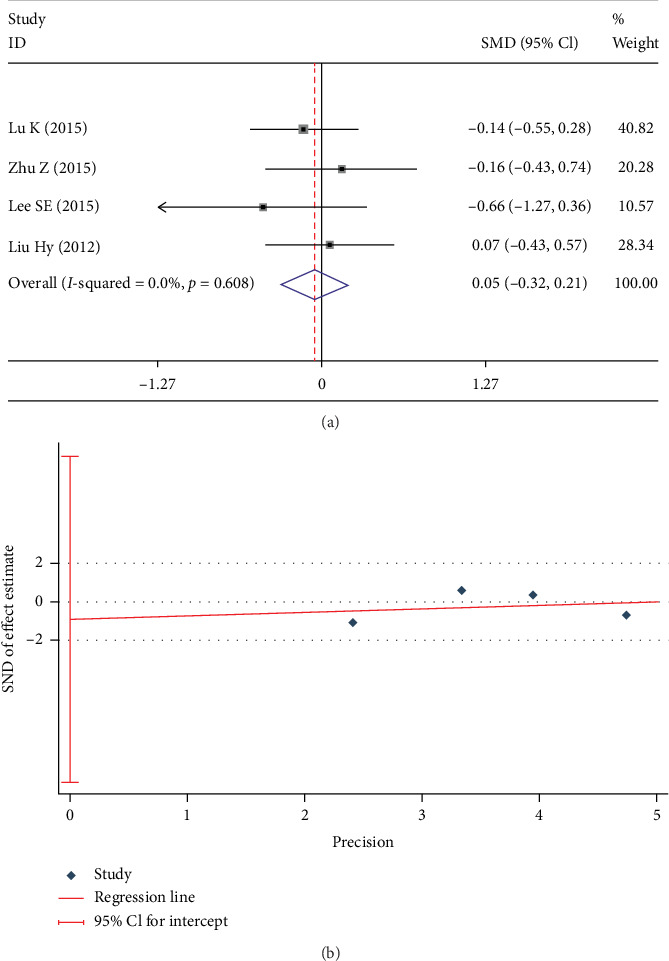
Test results showing (a) forest plot of LL, (b) Egger graph of LL.

LL was evaluated in 4 studies [8, 9, 20, 21]. No significant between-group difference was found in fixed-effects model (SMD -0.052; 95% CI -0.316, 0.211; *I*^2^ = 0%; *p* = 0.697, Figure 4(a)). No significant publication bias was found in the Egger test (*p* = 0.691, Figure 4(b)).

### 3.3. Comparison of the Clinical Outcomes

**Figure 5:**
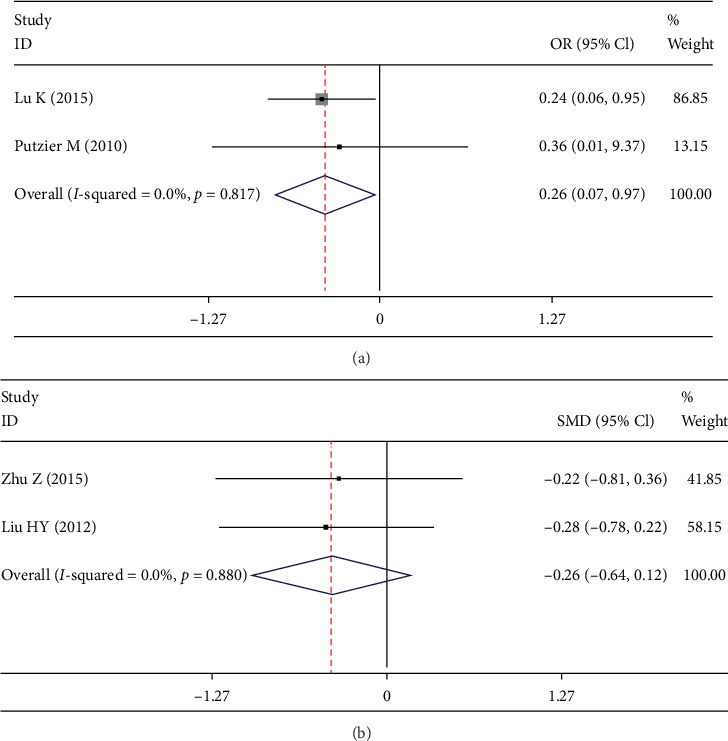
Forest plot showing (a) CASP, (b) JOA.

CASP refers to clinical symptoms related to RASP [13, 14]. We used fixed-effects model and found rate of CASP in DATF group was significantly less than PLIF group (OR 0.255; 95% CI 0.072, 0.911; *I*^2^ = 0%; *p* = 0.035, Figure 5(a)). JOA score was discussed in 2 studies [8, 9]. No significant between-group difference was found in fixed-effect model (SMD -0.256; 95% CI -0.635, 0.124; *I*^2^ = 0%; *p* = 0.186, Figure 5(b)).

**Figure 6:**
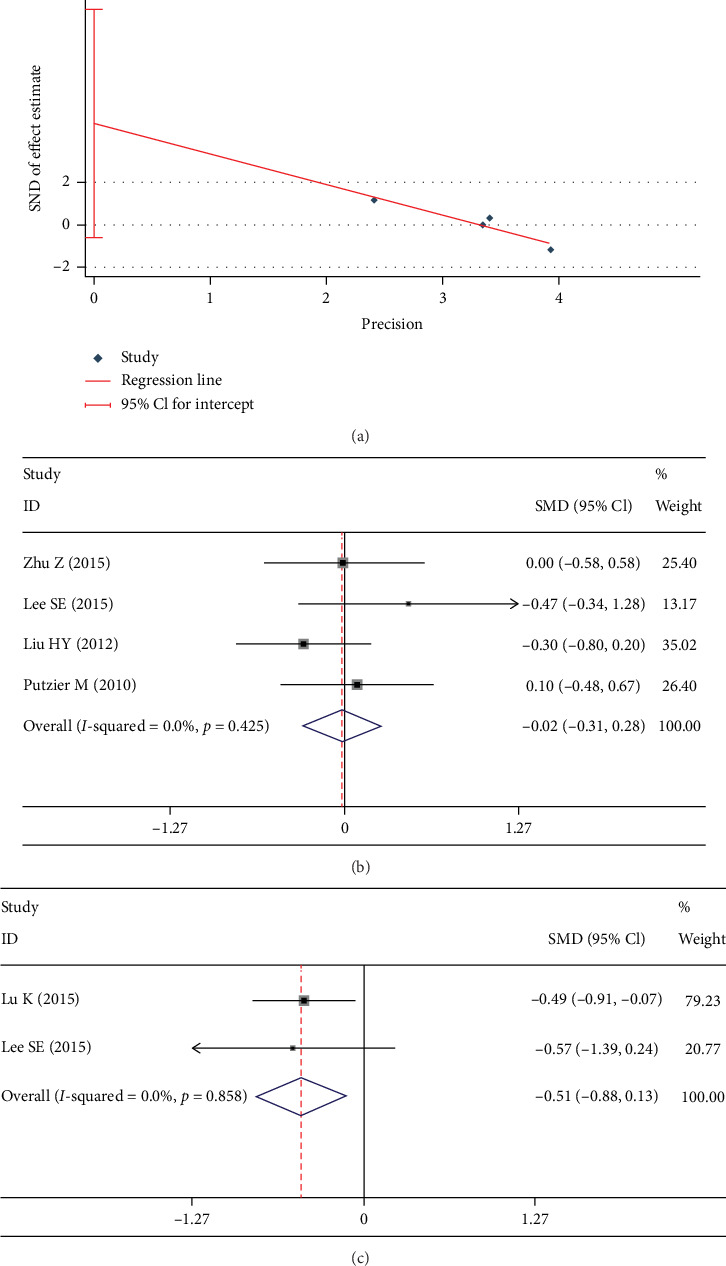
Test results showing (a) Egger graph of VAS-B, (b) forest plot of VAS-B, (c) forest plot of VAS-L.

VAS-B was reported in 6 studies [3, 8, 9, 20, 21, 23]. There was a significant heterogeneity in these studies (*I*^2^ = 54.8%). Egger test showed a significant publication bias (*p* = 0.001). The heterogeneity (*I*^2^ = 0%) and publication bias (*p* = 0.063) disappeared after the exclusion of two studies [3, 20] (Figure 6(a)). No significant between-group difference was found in fixed-effects model (SMD -0.018; 95% CI -0.313, 0.267; *I*^2^ = 0%; *p* = 0.903, Figure 6(b)). Two trials [20, 21], in both of which hybrid technology was used in the DATF group, evaluated VAS-L. We used the fixed-effects model and found VAS-L in the DATF group was significantly less than the PLIF group (SMD -0.506; 95% CI -0.879, -0.134; *I*^2^ = 0%; *p* = 0.008, Figure 6(c)).

**Figure 7:**
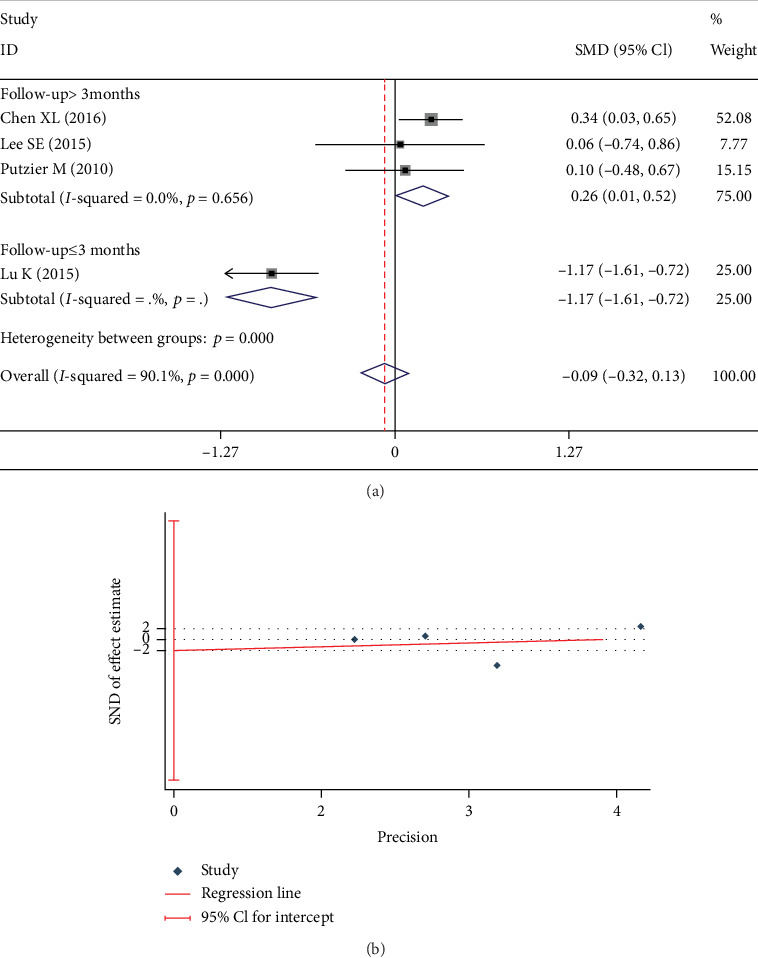
Test results showing (a) forest plot of ODI, (b) Egger graph of ODI.

Four studies [3, 20, 21, 23] evaluated postoperative ODI. There was a significant heterogeneity in the studies (*I*^2^ = 90.1%). We performed a subgroup analysis according to whether the follow-up was more than 3 months or not. There was no significant heterogeneity in each subgroup (*I*^2^ = 0%), which indicated the bias was caused by different follow-up. Analysis with fixed-effects model revealed significant between-group differences in postoperative ODI during 3 months follow-up (SMD -1.166; 95% CI -1.612, -0.719; *I*^2^ = 0%; *p* < 0.001) and after 3 months follow-up (SMD 0.264; 95% CI 0.006, 0.521; *I*^2^ = 0%; *p* = 0.045, Figure 7(a)). No significant publication bias was found in the Egger test (*p* = 0.745, Figure 7(b)).

### 3.4. Comparison of the Operative Parameters

**Figure 8:**
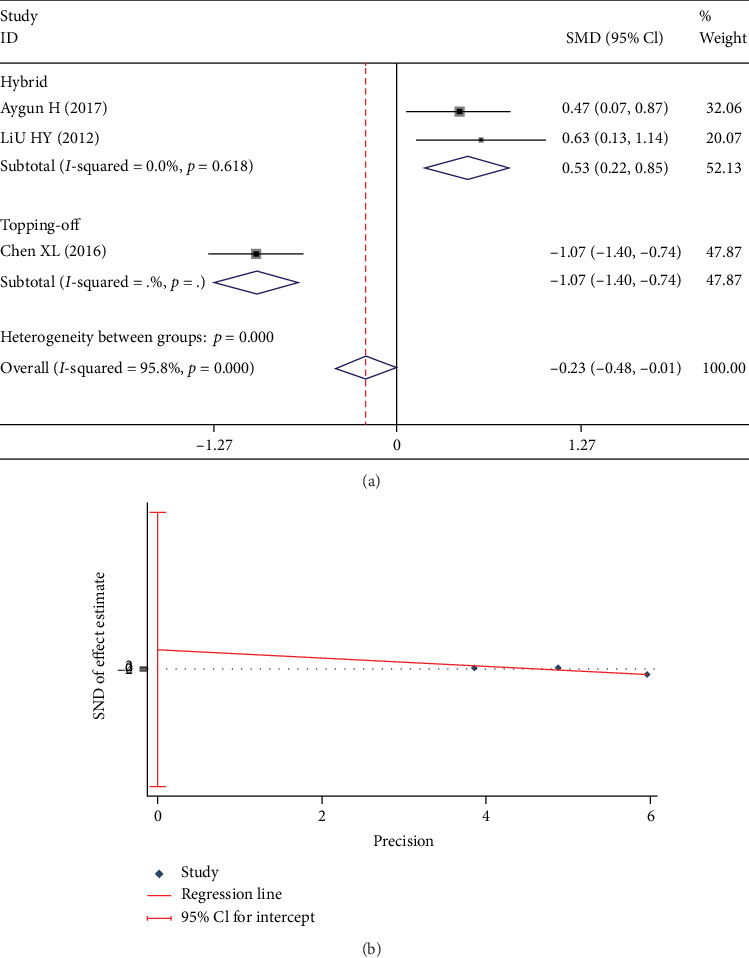
Test results showing (a) forest plot of DS, (b) Egger graph of DS.

Three studies [3, 9, 13] reported DS. There was a significant heterogeneity in these studies (*I*^2^ = 95.8%). A subgroup analyses was carried out in accordance with whether the DATF was hybrid technology or topping-off technology. The fixed-effect model was used for this analysis. No significant heterogeneity was found in each subgroup (*I*^2^ = 0%). It indicated that the bias was caused by different kinds of DATF. In hybrid subgroup, DS in DATF group was significantly larger than PLIF group (SMD 0.531; 95% CI 0.216, 0.845; *I*^2^ = 0%; *p* = 0.001, Figure 8(a)). However, in the topping-off subgroup, DS in the DATF group was significantly less than the PLIF group (SMD -1.068; 95% CI -1.396, -0.740; *I*^2^ = 0%; *p* < 0.001, Figure 8(a)). Egger test showed no significant publication bias (*p* = 0.324, Figure 8(b)).

**Figure 9:**
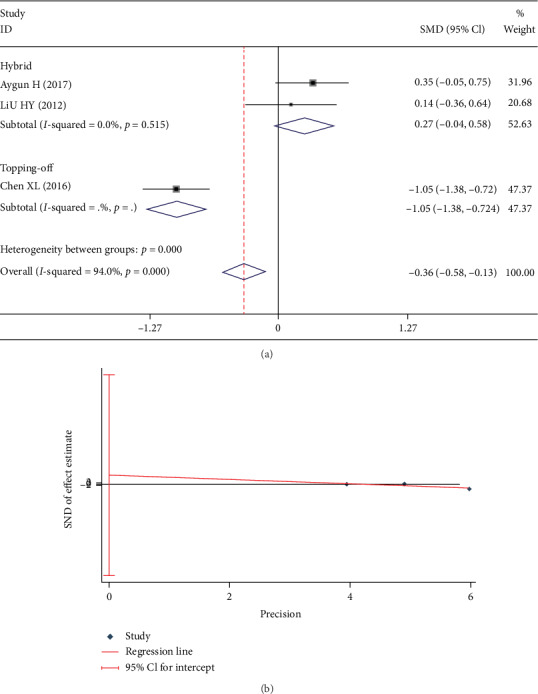
Test results showing (a) forest plot of EBL, (b) Egger graph of EBL.

EBL was estimated in 3 studies [3, 9, 13]. The fixed-effects model was used for the analysis and found a significant heterogeneity in these studies (*I*^2^ = 0%). We performed a subgroup analyses and divided these studies into hybrid subgroup and topping-off subgroup. There was no significant heterogeneity in each subgroup (*I*^2^ = 0%). It was confirmed that different kinds of DATF would cause the bias. No between-group difference was found in hybrid subgroup (SMD 0.268; 95% CI -0.043, 0.578; *I*^2^ = 0%; *p* = 0.091, Figure 9(a)). However, in the topping-off subgroup, EBL in the DATF group was significantly less than the PLIF group (SMD -1.049; 95% CI -1.377, -0.722; *I*^2^ = 0%; *p* < 0.001, Figure 9(a)). No significant publication bias was found in the Egger test (*p* = 0.430, Figure 9(b)).

### 3.5. Comparison of the Complications

**Figure 10:**
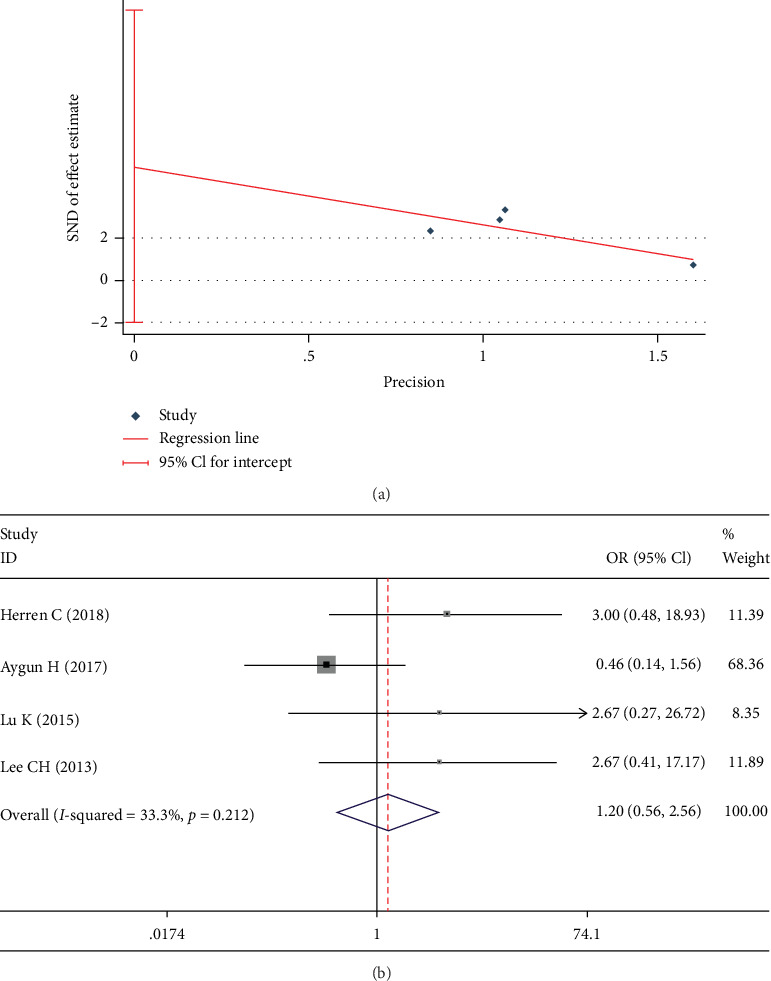
Test results showing (a) Egger graph of complication rate, (b) forest plot of complication rate.

The rate of complications was documented in 5 studies [13, 19–21, 23]. Egger test showed a significant publication bias in these studies (*p* = 0.048). The publication bias disappeared after the exclusion of one study [23] (*p* = 0.088, Figure 10(a)). No significant heterogeneity was found in these studies (*I*^2^ = 33.3%). There was no significant difference between the DATF group and PLIF group (OR 1.196; 95% CI 0.559, 2.560; *I*^2^ = 33.3%; *p* = 0.644, Figure 10(b)).

**Figure 11:**
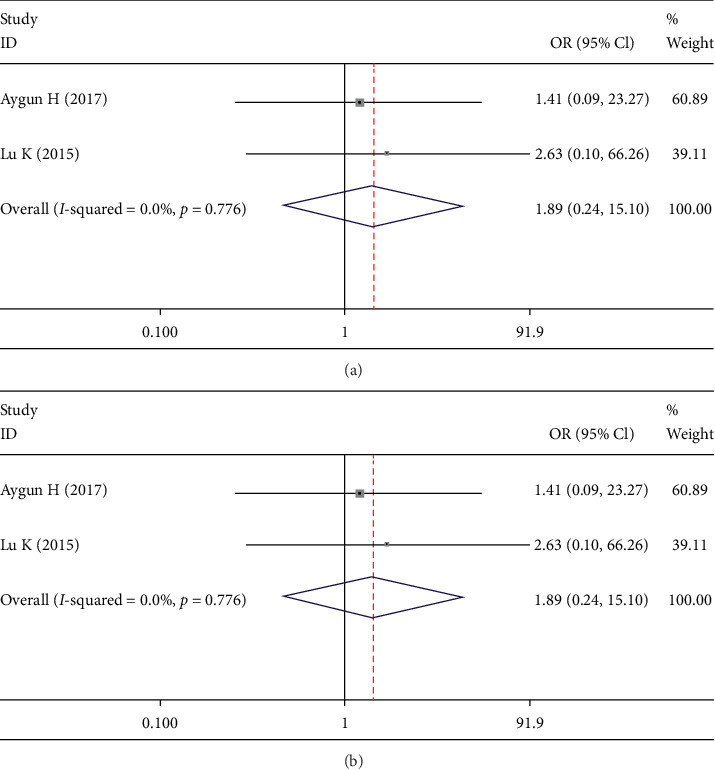
Forest plot showing (a) dural tear rate, (b) infection rate.

Two studies [13, 20] reported the rate of dural tear and infection. No between- group difference was found in the dural tear rate (OR 1.890; 95% CI 0.237, 15.095; *I*^2^ = 0%; *p* = 0.548, Figure 11(a)) and infection rate (OR 0.641; 95% CI 0.080, 5.115; *I*^2^ = 0%; *p* = 0.674, Figure 11(b)).

**Figure 12:**
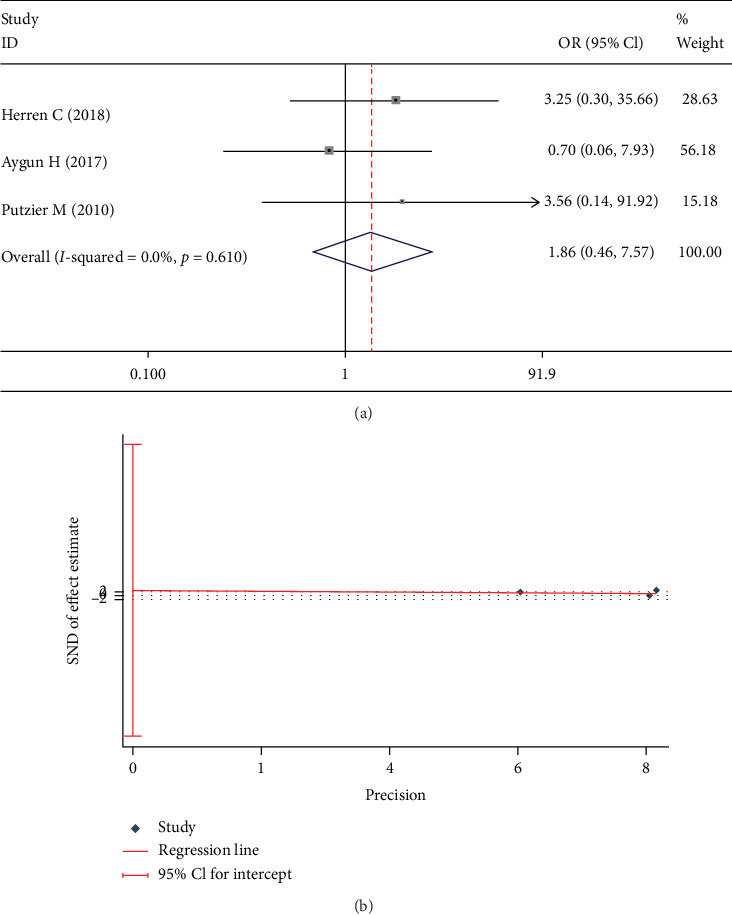
Test results showing (a) forest plot of implant loosening, (b) Egger graph of implant loosening.

The rate of implant loosening was documented in 3 studies [13, 19, 23]. No significant between-group difference was found in the fixed-effect model (OR 1.861; 95% CI 0.458, 7.573; *I*^2^ = 0%; *p* = 0.385, Figure 12(a)). Egger test showed no significant publication bias (*p* = 0.702, Figure 12(b)).

**Figure 13:**
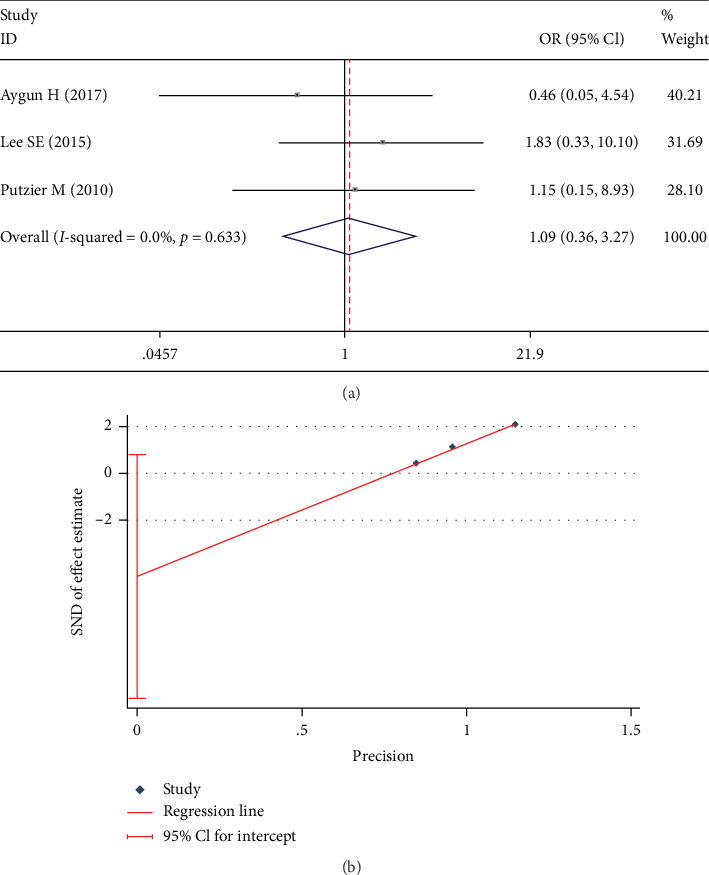
Test results showing (a) forest plot of pseudoarthrosis rate, (b) Egger graph of pseudoarthrosis rate.

The rate of pseudoarthrosis was reported in 3 studies [13, 21, 23]. The fixed-effects model was used for this analysis, and found no significant between-group difference (OR 1.087; 95% CI 0.362, 3.267; *I*^2^ = 0%; *p* = 0.882, Figure 13(a)). No significant publication bias was found in the Egger test (*p* = 0.059, Figure 13(b)).

**Figure 14:**
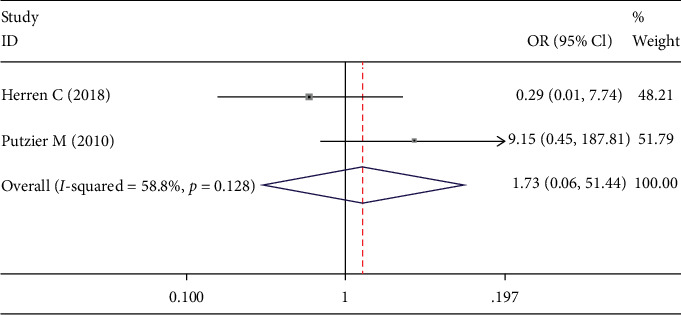
Forest plot showing implant breakage rate.

Data on the rate of implant breakage was available from 2 studies [19, 23]. There was a significant heterogeneity in these studies (*I*^2^ = 56.8%). Therefore, the random-effect model was used in this analysis. There was no significant difference between the DATF group and PLIF group (OR 1.734; 95% CI 0.058, 51.436; *I*^2^ = 56.8%; *p* = 0.750, Figure 14).

**Figure 15:**
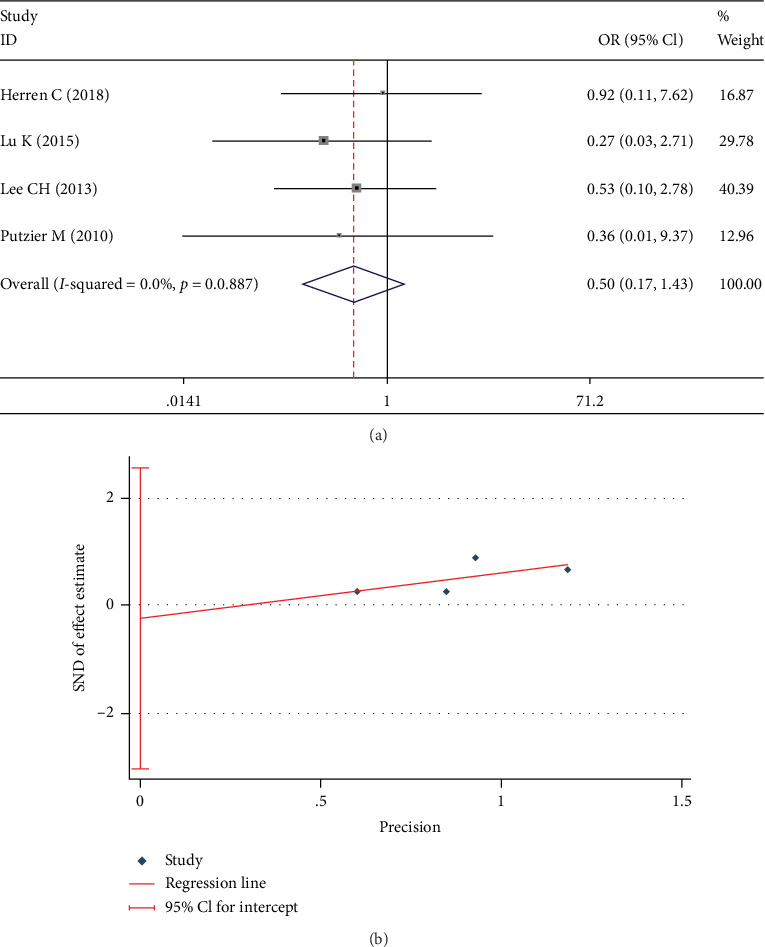
Test results showing (a) forest plot of reoperation rate, (b) Egger graph of reoperation rate.

Reoperation rate was documented in 4 studies [19, 20, 22, 23]. There was no significant heterogeneity in these studies (*I*^2^ = 0%). The fixed-effect model was used in this analysis and found no significant between-group difference in reoperation rate (OR 0.449; 95% CI 0.175, 1.427; *I*^2^ = 0%; *p* = 0.195, Figure 15(a)). Egger test showed no significant publication bias in these studies (*p* = 0.792, Figure 15(b)).

## 4. Discussion

Alternative DATF has been used to prevent the development of ASP after fusion surgery [24]. The definition of hybrid technology includes the application of two different kinds of devices, fusion, and dynamic stabilization. Hybrid technology is not to prevent progressive degeneration of the asymptomatic adjacent segment but is used to replace fusion in the treatment of symptomatic degenerated adjacent segments [6]. However, the topping-off technique combines rigid fusion with an interspinous process device in the adjacent segment in order to prevent ASD [8]. Therefore, there is a significant difference between the definitions of these two technologies. It is not precise to confuse the two concepts. In this study, we conducted a meta-analysis to tell the difference of outcomes between these two technologies compared with PLIF. Unfortunately, most studies did not make a clear distinction between the two concepts, which caused a great difficulty to our research. The results showed that the differences between hybrid surgery and topping-off technique were located in DS and EBL in comparison with PLIF. This indicated that, in most assessment methods, the treatment outcomes of the two technologies were similar.

A possible explanation for RASP is that when segments undergo fusion, the adjacent segments have to compensate for the most range of movement; this may cause the exposure of these segments to shear forces and overload [13]. Dynamic devices may disperse the loading of a facet by restriction both flexion and extension [25]. Therefore, the rate of proximal RASP in DATF was significantly less than PLIF in our research. In addition, our study showed that no difference was found in RASP distal to lumbar fusion between DATF and PLIF. The possible explanation is that DATF could increase stress on the lower adjacent segment; this may accelerate the long-term degeneration of the lower segment [26]. It was reported that the motion of fusion was not the only cause of RASP; the presence of spinal malalignment combined with fusion appeared to be a major factor resulting in RASP [13]. Considering our study showed that no significant between-group difference was found in LL, the preservation of RASP might not result from the realignment effect of DATF.

RASP and CASP are two different entities [27]. Clinical symptoms sometimes may not be observed in low grades of RASP, such as Grades 1 or Grade 2, and even in Grade 3 RASP [13]. Because there was still a lack of a suitable system for the clinical evaluation of ASP, CASP was evaluated as postoperative degenerative changes and related symptoms in adjacent segments. Previous studies discouraged the utilization of a pedicle screw-based dynamic stabilization system for the prevention of CASP [23, 28]. In our analysis, the study of Lu et al. [20] discussed the effect of DIAM; the range of motion of the proximal and distal adjacent segments was not significantly affected by DIAM implant. It was reported that the relatively young patient population without low grades of degeneration or need for decompression in the adjacent levels would not suffer from the development of CASP [7]. Although Putzier et al. [23] discussed Dynesys, which was a pedicle screw-based dynamic stabilization system, and the rate of CASP in DATF was lower than PLIF in their research. The possible explanation was that the average age of their patients was relatively young. Therefore, our results showed that the rate of CASP was lower in DATF than PLIF.

Our study showed that there was no significant difference between DATF and PLIF in postoperative JOA and VAS-B; the ODI in DATF was significantly lower than PLIF during 3 months follow-up and then a rebound occurred. These implied that DATF might have a minimal influence on the functional outcome. Our study showed that VAS-L in DATF was significantly less than PLIF. This might be partially explained by the leg pain that was related to lumbar radiculopathy in the fusion segments rather than the adjacent segments [22].

Our results showed that DS in hybrid surgery was significantly larger than PLIF; however, DS in topping-off techniques was significantly less than PLIF. In addition, no significant difference between hybrid surgery and PLIF was found in EBL; nevertheless, EBL in DATF was significantly less than PLIF. This was because topping-off techniques were usually used in combination with short-segment fixation, while hybrid surgery was more complexed than others [29].

The potential of DATF in decreasing the incidence of complications after fusion surgery remains controversial. Putzier et al. [23] pointed out that the forces conveyed from the dynamic implant could increase the stress on rigid fixation over time, and then implant-associated adverse events would occur. However, our study showed that no significant difference between DATF and PLIF was found in the rate of complications, such as dural tear, infection, implant loosening, pseudoarthrosis, and implant breakage; in addition, there was no significant difference between DATF and PLIF in the reoperation rate. These implied that the observed complications were all independent of the implanted instrumentation. Even though Bredow et al. [30] performed a review on this topic, they failed to run a data analysis to come to a persuasive conclusion.  Table 2 showed that the possible bias caused by the included articles was representativeness of the cases and exposure. Considering the random-effect models were applied unless statistical heterogeneity was insignificant, in which case fixed-effect models were used in this research. Through subgroup analysis, the influence of study design and fixed levels on pooled estimates was investigated by us. In addition, the Egger test was used to analyze the publication bias. The fixed-effect model was used for almost all comparisons in this study. In addition, all the factors leading to bias have been well quantified. It can be concluded that this study is a meta-analysis with a level III of evidence. Therefore, our results were more convincing. Although TLIF is widely used to treat degenerative diseases of the lumbar spine, most of the comparisons between hybrid surgery and traditional fusion surgery in the previous studies discussed the topping-off technique with the PLIF technique. This makes us lack of literature support when discussing TLIF surgery. In addition, PLIF surgery can seriously damage the posterior column structure, so it is suitable for hybrid surgery [31]. However, relatively speaking, TLIF surgery has less damage to the stable structure of the posterior column [32]. Therefore, previous studies did not consider the use of hybrid surgery for further treatment after TLIF surgery. Therefore, TLIF surgery is not the focus of this study.

## 5. Conclusion

The difference between hybrid surgery and topping-off technique was located in DS and EBL in comparison with PLIF. The difference between these two techniques was not significant in treatment outcomes. DATF was better than PLIF in proximal RASP, CASP, and ODI during 3 months follow-up, VAS-L. However, no significant difference between DATF and PLIF was found in distal RASP, LL, JOA score, VAS-B, ODI after 3 months follow-up, complication rates, and reoperation rate. These further confirmed that DATF could decrease the proximal ASP both symptomatically and radiographically as compared to the fusion group; however, the influence of DATF on functional outcome was similar with PLIF. More high-quality researches are required to confirm whether DATF is better than PLIF in the treatment of lumbar degenerative disease.

## Figures and Tables

**Table 1:** Characteristics of included studies.

Study	Year	Country	Study type	LoE	Device	Patients (F/M)		Age (years)		Follow-up (months)	Segments	Parameters	Complications
						PLIF	DATF	PLIF	DATF				
Herren et al. [19]	2018	Germany	RCT	I	Dynesys	14 (8/6)	15 (6/9)	61.78 (34-76)	60.9 (47-80)	37.68 (1.38-72)	1-5	NA	Implant loosening, implant breakage
Aygun et al. [13]	2017	Turkey	Retro	III	Cosmic	59 (34/25)	42 (19/23)	54.2 ± 5.11	52 ± 6.02	79	1-5	RASP, EBL, DS	Dural tear, radiculopathy, infection, implant loosening, Pseudoarthrosis
Chen et al. [3]	2016	China	Retro	III	Coflex	88 (34/54)	76 (28/48)	58.31 ± 4.6	57.34 ± 5.1	47.2	1	RASP, VAS, ODI, EBL, DS	NA
Lu et al. [20]	2015	China (Taiwan)	Retro	III	DIAM	42 (14/28)	49 (16/33)	64.5 ± 7.2	59.1 ± 8.6	41.5	2-4	RASP, CASP, LL, VAS, ODI	Dural tear, infection, deep vein thrombosis, acute myocardial infarction
Zhu et al. [8]	2015	China	Retro	III	Wallis	23 (12/11)	22 (8/14)	40	44.5	24	1	VAS, JOA	NA
Lee et al. [21]	2015	Korea	Retro	III	DTO/Nflex	10 (5/5)	15 (11/4)	63.9 ± 7.8	60.7 ± 8.3	48	2	RASP, LL, VAS, ODI	Pseudoarthrosis
Lee et al. [22]	2013	Korea	Retro	III	DIAM	50 (20/30)	25 (10/25)	65.9 ± 8.5	65.4 ± 8.7	46.8	1	RASP	NA
Liu et al. [9]	2012	China	Retro	III	Coflex	48 (20/28)	31 (11/20)	41.5	44.6	24	1	LL, VAS, JOA, EBL, DS	NA
Putzier et al. [23]	2010	Germany	Pro	II	Dynesys	30 (16/14)	30 (13/17)	44.6	44.9	76.4	1	RASP, CASP, VAS, ODI	Implant loosening, pseudoarthrosis, implant breakage

**Table 2:** Newcastle-Ottawa scale (NOS).

Items	Scales								
Categories	Herren et al. [19]	Aygun et al. [13]	Chen et al. [3]	Lee et al. [21]	Lu et al. [20]	Zhu et al. [8]	Lee et al. [22]	Liu et al. [9]	Putzier et al. [23]
Selection									
(1) Is the case definition adequate?	1	1	1	1	1	1	1	1	1
(a) Yes, with independent validation.									
(b) Yes, e.g., record linkage or based on self-reports.									
(c) No description.									
(2) Representativeness of the cases.	1	0	1	1	1	0	1	1	1
(a) Consecutive or obviously representative series of cases.									
(b) Potential for selection biases or not stated									
(3) Selection of controls.	1	1	1	1	1	1	1	1	1
(a) Community controls.									
(b) Hospital controls.									
(c) No description.									
(4) Definition of controls.	1	1	1	1	1	1	1	1	1
(a) No history of disease (endpoint).									
(b) No description of source.									
Comparability									
(1) Comparability of cases and controls on the basis of the design or analysis.	2	2	2	2	2	2	2	2	2
(a) Study controls for topping-off technique.									
(b) Study controls for any additional factor.									
Exposure									
(1) Ascertainment of exposure.	1	0	0	1	0	0	0	0	0
(a) Secure record (e.g., surgical records).									
(b) Structured interview where blind to case/control status.									
(c) Interview not blinded to case/control status.									
(d) Written self-report or medical record only.									
(e) No description.									
(2) Same method of ascertainment for cases and controls.	1	1	1	1	1	1	1	1	1
(a) Yes.									
(b) No.									
(3) Non-response rate.	1	0	0	0	0	0	0	0	0
(a) Same rate for both groups.									
(b) Non respondents described.									
(c) Rate different and no designation.									
Total	9	6	7	8	7	6	7	7	7

## Abbreviations

LBP: Low back pain
HSD: Hybrid stabilization device with pedicle screw or rod construct
IPD: Interspinous process devices
TFR: Total facet replacement system
ASDeg: Adjacent segment degeneration
ASDis: Adjacent segment disease
DATF: Dynamic stabilization adjacent to fusion
PLIF: Posterior lumbar interbody fusion
PRISMA: Preferred reporting items for systematic reviews and meta-analyses
RCTs: Randomized controlled trials
CASPs: Clinical adjacent segment pathologies
RASPs: Radiological adjacent segment pathologies
LL: Lumbar lordosis
VAS: Visual analogue scale
VAS-B: VAS of back
VAS-L: VAS of leg
ODI: Oswestry disability index
JOA: Japanese Orthopaedic Association
DS: Duration of surgery
EBL: estimated blood loss.
NOS: Newcastle-Ottawa scale
LoE: Level of evidence
OR: Odds ratios
CI: Confidence interval
WMD: Weighted mean differences.
